# Rapid Fatal Outcome of Cryptococcal Meningoencephalitis in a Non-HIV Immunocompromised Patient with a Low Fluconazole Susceptibility Isolate: A Case Report from Madagascar

**DOI:** 10.1155/2016/3492504

**Published:** 2016-12-18

**Authors:** Mihaja Raberahona, Rivonirina A. Rakotoarivelo, Njary Randriamampionona, Angelot F. Rakotomalala, Tiana Razafinambinintsoa, Thomas Bénet, Philippe Vanhems, Mamy J. D. D. Randria, Muriel Cornet, Mala Rakoto-Andrianarivelo

**Affiliations:** ^1^Service des Maladies Infectieuses, HU Joseph Raseta de Befelatanana, Antananarivo, Madagascar; ^2^Service des Maladies Infectieuses, CHU Tambohobe, Fianarantsoa, Madagascar; ^3^Centre d'Infectiologie Charles Mérieux, Antananarivo, Madagascar; ^4^Service d'Hygiène, Epidémiologie et Prévention, Hospices Civils de Lyon, Lyon, France; ^5^Laboratoire des Pathogènes Emergents, Fondation Mérieux, Centre International de Recherche en Infectiologie (CIRI), INSERM U1111, CNRS UMR5308, ENS de Lyon, UCBL1, Lyon, France; ^6^Laboratoire de Parasitologie-Mycologie, Institut de Biologie et de Pathologie, CHU Grenoble Alpes, Grenoble, France; ^7^Laboratoire TIMC-IMAG-TheREx, Université Grenoble-Alpes, Grenoble, France

## Abstract

Cryptococcal meningoencephalitis is considered rare in HIV-negative individuals. In Madagascar, the epidemiology of cryptococcosis has not yet been well described, neither in immunocompetent nor in immunocompromised patients. We report here the first Malagasy detailed case of cryptococcal meningoencephalitis in a non-HIV immunocompromised adult patient carrying a low fluconazole susceptibility isolate. We emphasize the importance of early and accurate diagnosis to meet the challenges of managing cryptococcosis in developing countries.

## 1. Introduction

Cryptococcosis is mostly caused by two encapsulated yeasts,* Cryptococcus neoformans* and* Cryptococcus gattii*. It is a life-threatening infection occurring mainly in patients with impaired cellular immunity with >1,000,000 cases estimated each year worldwide [1]. Sub-Saharan Africa has the highest incidence and mortality attributed to cryptococcal meningoencephalitis (CM) with more than 600,000 deaths reported each year among HIV/AIDS patients [2]. By contrast, in developed countries, the overall incidence of cryptococcosis in HIV/AIDS patients has been decreasing due to the improved access to antiretroviral therapy. In these regions, the burden of the disease appears to be increasing in non-HIV-immunocompromised populations [3]. In countries where the incidence of HIV remains high, investigating CM is paramount whenever the CD4 count drops below 100/mm^3^. However, cryptococcosis does not necessarily come to mind in an apparently immunocompetent individual. Depending on the site of invasion of the pathogen, a disseminated cryptococcal infection can be manifested in various clinical forms that include meningoencephalitis, pulmonary disease, skin disease, osteomyelitis, peritonitis, or comorbidities [4–9]. In Madagascar, little is known on the epidemiology or the clinical presentations of cryptococcosis. Here we report a rapid fatal outcome of meningoencephalitis due to* Cryptococcus neoformans* var.* grubii* in a patient without known immunosuppression history. The importance of rapid and accurate diagnosis of cryptococcosis is emphasized. As the isolate showed a low level of fluconazole susceptibility, therapeutic challenges faced in the management of patients in low income countries are also discussed.

## 2. Case Report

A 55-year-old farmer, male, was admitted to the Intensive Care Unit of the Befelatanana University Hospital in Antananarivo with an altered consciousness. One month prior to his admission, he complained of a moderate and diffuse headache associated with blurred vision. His general condition worsened quickly during the 3 days prior to admission with the onset of fever and confusion. At admission, physical examination revealed a mild fever (38.6°C), but most of the other physiological parameters were within normal limits. Glasgow Coma Scale (GCS) was 4/15; the patient presented unresponsive bilateral tight myosis and signs of meningeal irritation, that is, neck rigidity, Kernig, and Brudzinski signs. No focal neurological signs were observed. Chest auscultation revealed crackles of the lower zones on both sides and diffuse rhonchi. A lumbar puncture showed a high opening pressure (35 cm H_2_O) of cerebrospinal fluid (CSF), which was macroscopically limpid. Examination of other systems revealed no apparent abnormalities, and there was no history of long-term medication with corticosteroids, solid organ or stem transplantation, hematologic malignancies, or chronic disease conditions, such as tuberculosis or kidney/hepatic failure.

Initial laboratory investigations of the CSF showed normal white count and glucose and an increased concentration of protein at 288 mg/dL (normal: <40 mg/dL). Blood count showed elevated white cells at 14 × 10^3^/*μ*L (normal: 4.4 to 10.5 × 10^3^/*μ*L) with 91% neutrophils (normal: 42 to 77%) and 7% lymphocytes (normal: 17 to 49%). Other biological parameters were within normal ranges. A rapid HIV serological test (Alere Determine™ HIV-1/2, Alere Inc, USA) was negative; RNA HIV-1 viral load (Generic HIV Charge Virale, Biocentric, Bandol, France) was undetectable, and CD4 count (Alere Pima™ CD4, Ireland) was 126 cells/mm^3^. The brain Computerized Tomography scan and chest radiography were normal.

**Figure 1:**
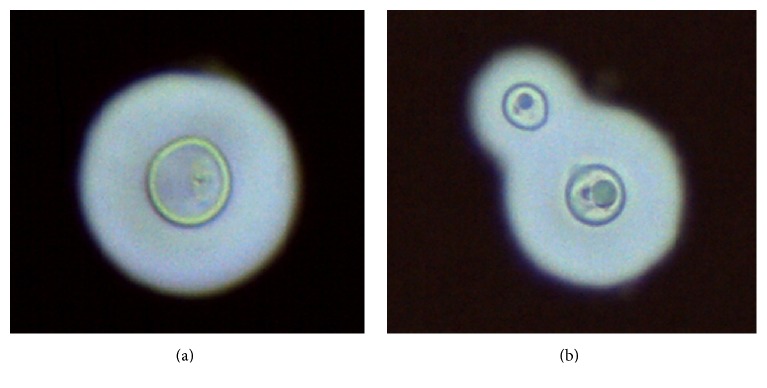
Microscopy of the* Cryptococcus neoformans* isolates in the CSF stained with India ink showing spherical (a) and budding yeasts (b) surrounded by a clear haloed zone (×400).

Direct microscopic examination of CSF with India ink showed the presence of a large number of spherical yeasts surrounded by a clear haloed zone consistent with* Cryptococcus* spp. (Figure 1). A lateral-flow immunochromatographic assay for the rapid and semiquantitative detection of* Cryptococcus* antigen (CrAg® LFA Immuno-Mycologics, Inc., OK, USA) [10] revealed a low titre (1 : 40) in the CSF and a higher titre (1 : 160) in the serum. Subsequently, the CSF, inoculated onto Sabouraud-chloramphenicol media without cycloheximide, incubated at 30°C, yielded smooth yeast colonies later confirmed as* Cryptococcus neoformans* var.* grubii* by matrix-assisted laser desorption ionization time-of-flight (MALDI-ToF) mass spectrometry using Bruker BioTyper instrument. The isolate was correctly identified at the species and varietal level with a log (score) value of spectra of 2.12. The antifungal susceptibility of the isolate was tested retrospectively by using *E*test (BioMérieux, La Balme les Grottes, France). The Minimum Inhibitory Concentration (MIC) was determined as the lowest concentration that inhibited 100% and 80% of the fungal growth for amphotericin B and azoles, respectively. The MIC value for amphotericin B was 0.25 *μ*g/mL, for voriconazole 0.38 *μ*g/mL, and for fluconazole 48 *μ*g/mL. The high MIC value of fluconazole was confirmed at 32 *μ*g/mL by the EUCAST reference method (EDef 7.2 Revision) [11]. Following antigen detection and direct examination of CSF, the patient was treated immediately with fluconazole (1200 mg/day)* via* nasogastric tube, but the outcome was fatal after 7 days of treatment with acute respiratory distress and worsening of the neurological status with transition to unresponsive coma.

## 3. Discussion

Meningoencephalitis is the most common manifestation of cryptococcal disease and is mainly caused by* C. neoformans* in immunocompromised patients. In this case, we isolated* C. neoformans* var.* grubii*, a pathogen distributed worldwide, capable of invading the central nervous system, especially during an HIV infection, the main risk factor for the development of CM. We suspected CM, because the patient presented with headache, fever, and altered consciousness. However, HIV antibodies were not detected and the patient's history ruled out other causes of immunosuppression aside from a low CD4 cell count (126 cells/mm^3^). Unexplained opportunistic infections such as CM have been associated with idiopathic CD4 lymphopenia in HIV-uninfected patients [12, 13], but in our case the immune response has not been investigated. It is also likely that the CD4 count may have been low because the specimen was taken during an acute illness, rather than being the underlying cause of immunosuppression.

By using the rapid, easy, and point-of-care CrAg LFA test,* Cryptococcus* spp. infections should no longer be overlooked by clinicians in developing countries. In our laboratory, the cost of diagnosis based on CrAg LFA was estimated at around 5.60 € per test. Our case shows that culturing the pathogen remains crucial for both species' identification (*C. neoformans versus C. gattii*) and antifungal susceptibility testing despite the lack of interpretative breakpoints. WHO recommends treating immunocompromised patients with CM with a combination of amphotericin B and flucytosine for two weeks followed by 8–10 weeks of fluconazole (400–800 mg daily) and then fluconazole (200 mg daily) for an additional 6–12 months [14]. If amphotericin B and flucytosine are unavailable, as it is the case in Madagascar, a two-week induction phase of fluconazole (1200 mg daily) followed by an 8-week consolidation phase of fluconazole (800 mg daily) is recommended. Soon after diagnosis, our patient was treated with fluconazole (1200 mg daily). A high titre of CSF CrAg is commonly associated with very poor prognosis, but in our case the low titre observed (1 : 40) suggests that other factors were involved, including delayed presentation to the hospital, altered mental status with very low GSC score, suboptimal induction phase antifungal treatment, and high CSF opening pressure, which is frequently associated with high mortality during the course of CM [15]. The observed high MIC of* C. neoformans* to fluconazole may have also played a role in the outcome of the disease, as well as a highly neurovirulent* C. neoformans* strain or intrinsic immunological host factors. However, these last two factors were not explored in this study. In developing countries, challenges to facilitate access to more effective molecules to treat patients with CM have to deal with complicated administrative formalities for the import of medication and a weak national HIV control programme.

In conclusion, a cryptococcal infection should be considered in all individuals presenting with meningoencephalitis, where there is possible immunodeficiency involvement and no known risk factors. We recommend that individuals presenting with meningoencephalitis and low CD4 cell count with or without risk factors for HIV infection be thoroughly examined for possible aetiology of cryptococcosis. Standardized rapid diagnostic tests, for example, CrAg LFA, should become routine. Fluconazole low susceptibility may be encountered highlighting the need for more effective alternatives for treatment to reduce mortality in developing countries. A study is underway to assess the prevalence of CM in HIV-infected patients.
